# Potential Roles of PTEN on Longevity in Two Closely Related *Argopecten* Scallops With Distinct Lifespans

**DOI:** 10.3389/fphys.2022.872562

**Published:** 2022-07-12

**Authors:** Hanzhi Xu, Xia Lu, Chunde Wang, Junhao Ning, Min Chen, Yuan Wang, Ke Yuan

**Affiliations:** ^1^ Research and Development Center for Efficient Utilization of Coastal Bioresources, Yantai Institute of Coastal Zone Research, Chinese Academy of Sciences, Yantai, China; ^2^ College of Marine Science and Engineering, Qingdao Agricultural University, Qingdao, China; ^3^ University of Chinese Academy of Sciences, Beijing, China

**Keywords:** bivalve, *Argopecten* scallops, PTEN, nutrition restriction, genomic stability, PI3K/Akt/FoxO

## Abstract

Phosphatase and tensin homolog deleted on chromosome ten (PTEN) has been found to regulate longevity through the PI3K/Akt/FoxO pathway and maintenance of genome integrity in worms, flies, and mammals. However, limited information is available on the roles of PTEN in longevity of aquatic animals. Here we extended this paradigm using two closely related *Argopecten* scallops, *Argopecten purpuratus*, and Argopecten *irradians*, with significantly distinct life spans, which are commercially important bivalve species for fishery and aquaculture in China, United States, Peru, and Chile. The ORFs of the *ApPTEN* and *AiPTEN* were 1,476 and 1,473 bp, which encoded 491 and 490 amino acids, respectively. There were 48 synonymous and 16 non-synonymous SNPs and one InDel of three nucleotides between *ApPTEN* and *AiPTEN*, resulting in variations in 15 amino acids and lack of S453 in AiPTEN. Differences in conformation and posttranslational modification were predicted between ApPTEN and AiPTEN, which may indicate different activities of ApPTEN and AiPTEN. When the animals were subjected to nutrition restriction, the expression of both *ApPTEN* and *AiPTEN* was upregulated, with *AiPTEN* responded faster and more robust than *ApPTEN*. Ionizing radiation induced significantly elevated expression of *ApPTNE* but not *AiPTEN* in the adductor muscle, and the mortality rate of *A. purpuratus* was significantly lower than that of *A. irradians*, indicating that *ApPTNE* may play a protective role by maintaining the genome integrity. RNAi of *ApPTNE* significantly downregulated the expression of its downstream regulated genes known to favor longevity, such as *FoxO*, *Mn-SOD*, and *CAT*. These results indicated that PTEN may contribute to the longevity of *A. purpuratus* through regulation of nutrient availability and genomic stability, probably *via* PI3K/Akt/FoxO pathway. Our study may provide new evidence for understanding of the conservative functions of PTEN in regulation of lifespan in animals and human, and it may also benefit the selection of scallops strains with long lifespan and thus larger size.

## Introduction

Aging and longevity remain a mystery of biology, and the fundamental mechanism has been a worldwide hot issue for decades. The studies in model organisms have shown that both environmental and genetic factors influence life spans (Gladyshev, 2016). Nutrient level, temperature, and exogenous stimuli are the main environmental factors, and the functions of insulin/IGF-1 signaling pathway (IIS) in aging are highly conserved in evolution from *Caenhorhabditis elegans* to mammals (Kenyon, 2010). A number of studies reported that caloric restriction (CR) may extend longevity by decreasing activity of the IIS signaling axis (Fontana et al., 2010; Kenyon, 2010), and PI3K/Akt cascade is the core signaling axis of IIS pathway (Sun et al., 2017). Tumor suppressors such as the phosphatase and tensin homolog deleted on chromosome ten (PTEN) are well known for their ability to protect organisms from cancer and damages that favor aging (Cristofano et al., 1998; Ortega-Molina and Serrano, 2013; Chen et al., 2018). The most prominent function of PTEN is to inhibit the activity of PI3K/Akt signaling pathway through dephosphorylation of phosphatidylinositol-3,4,5-trisphosphate (PI(3,4,5)P3) (Carracedo and Pandolfi, 2008). Inhibition of PI3K/Akt induces the inactivation of FoxO transcription factors that play crucial roles in anti-aging and longevity (Nakamura et al., 2000; Sun et al., 2017). It was revealed that the high expression of FoxO could extend lifespan in the model organisms (Martins et al., 2016; Tia et al., 2018). As the unique negative regulator of PI3K/Akt/Foxo cascade pathway, the function of PTEN in lifespan regulation has attracted much attention in recent years. In the nematode *Caenorhabditis elegans*, both decreased activity of PI3K (AGE-1) and increased expression of PTEN (DAF-18) result in extended longevity (Dorman et al., 1995; Morris et al., 1996; Masse et al., 2005). In mice, PTEN^tg^ mice carrying additional genomic copies of PTEN are protected from cancer and exhibited a significant extension of life span (Ortega-Molina et al., 2012). CR as a robust antiaging intervention (Fontana et al., 2010) was also found to be associated with reduced basal activities of PI3K/Akt pathway (Li et al., 2008; Hempenstall et al., 2010; Moore et al., 2010). Overexpression of PTEN significantly inhibited cell migration and cell-cycle progression and promoted apoptosis in PCA cells by decreasing Ccnd1 expression and increasing that of Cdkn1b, which ultimately prolonged the life span of TRAMP mice (Ai et al., 2020). In human, Kirstein et al., (2021) found that cells with knockout PTEN showed less senescence compared with controls, and the senescence marker CDKN1A (p21) was downregulated in cells with knocked-down PTEN. In addition, previous studies implicated PTEN played roles in maintenance of genome integrity for longevity (Ho et al., 2020). Maintenance of genomic stability requires DNA repair mechanisms to protect DNA from genotoxic agents such as reactive oxygen species (ROS), ionizing radiation (IR) or ultraviolet radiation. PTEN loss was found to be associated with an accumulation of DNA double-strand breaks (DSBs), leading to a defect in DNA DSB repair (Bassi et al., 2013). PTEN regulated the expression of Rad51, a critical component of the DSB DNA repair pathway (He et al., 2016). It was also found that PTEN plays a direct role in the DNA damage response (DDR), as cells lacking PTEN were found to be sensitive to genotoxic stresses (Bassi et al., 2013).

Existing studies on aging and longevity mainly focused on several terrestrial model organisms such as the fruit flies, nematode worms, and mice, but with only a few reports in aquatic animals. Some scholars suggested that bivalves may be more appropriate for aging and longevity studies which may help explore the genetic basis of the lifespan in higher animals (Estabrooks, 2007; Abele et al., 2009). Especially, those taxonomically closely related bivalves with distinct life spans, such as the Peruvian scallop (*Argopecten purpuratus*) and bay scallop (*A. irradians*), may be used as alternative novel model organisms for studies on longevity, as it may help to elucidate how some key genes modify the lifespan in these animals. The Peruvian scallops and the bay scallops are commercially important bivalve species for fishery and aquaculture in China, United States, Peru, and Chile (Wang et al., 2009; Mendo et al., 2016). Although *A. purpuratus* and *A. irradians* diverged from a common ancestor probably in late Miocene, they have evolved into distinct lifespans during long-term adaptation to different natural environments (DiSalvo et al., 1984). *A. purpuratus* separated from the Atlantic stock *A. irradians* along with the closing of the Atlantic-Pacific connection at the end of the Miocene (Waller, 1969). After separating as a result of geographical isolation, *A. purpuratus* evolved into a cold-water species with a lifespan of 7–10 years (DiSalvo et al., 1984; Estabrooks, 2007; Wang et al., 2009), whereas *A. irradians* evolved into a warm water species with a lifespan of less than 2 years (Waller, 1969; Barber and Blake, 1983). Thus, the two *Argopecten* scallops may provide a unique opportunity for the study to determine how the key genetic factors such as PTEN gene define the longevity in so closely related species.

In the present study, we cloned and characterized the PTEN genes and investigated their functions in longevity in the two closely related *Argopecten* scallops with significantly distinct life spans. The results would provide new evidence for understanding the conservative function of PTEN in animals and eventually benefit scallop breeding.

## Materials and Methods

### Animals

The Peruvian scallop (*A. purpuratus*) and bay scallop (*A. irradians*) were cultured in open sea located in Yangma Island of Yantai, Shandong Province, China. They were brought into the scallop hatchery of Yantai Spring-Sea AquaSeed Co., Ltd. located in Laizhou, Yantai, Shandong province of China in early spring and conditioned to mature. They were induced to spawn individually in 1-L beakers after exposing to air for 30 min. The spawning scallops were watched carefully to collect eggs and sperm separately. Eggs or sperm from different individuals of the same species were pooled together. Then sperm were mixed with eggs to obtain fertilized eggs for production of the next generation. About 10 days after fertilization, when about 50% larvae developed eyespots, they were set on plastic collectors. After metamorphosis, the juveniles were moved to a shrimp pond for nursery for a period of 1 month and then to the open sea for another month before they were dispersed into lantern nets for grow-out. After about 9-months of cultivation, the scallops were collected for subsequent experiments.

### Cloning of the cDNA of *ApPTEN* and *AiPTEN*

Total RNA of the muscle tissue was extracted by Trizol reagent (TaKaRa, Kusatsu, Japan) and phenol chloroform method according to the manufacturer’s protocols. The purity and concentration of the total RNA were determined by Nanodrop 2000 spectrophotometer (Nano Drop Technologies, United States), and RNA integrity was detected by electrophoresis on 1.5% agarose gel electrophoresis. The complementary DNA (cDNA) for gene cloning was synthesized from total RNA (1 μg) using PrimeScript RT reagent kit (TaKaRa, Japan) following the manufacturer’s protocol. Then these products were stored at −20°C until the next step.

The CDS sequences of PTEN from the whole genome sequence of *A. purpuratus* (Li et al., 2018) and *A. irradians* (Liu et al., 2020) were used as the references to design primers for amplifying the cDNA of PTEN for the two species. Primers were then designed based on these sequences for amplification of the complete ORF cDNAs of PTEN from the two species (Table 1). PCR reactions were conducted using first-strand cDNA samples from mixed muscle tissues of five animals as template, and Premix Taq™ (TaKaRa, Japan) was used for the PCR reactions following the manufacturer’s protocol. All amplified DNA fragments were subcloned into a pMD19 PCR cloning vector (Sangon, China) for sequencing in Sangon Biotech (Qingdao) Co., Ltd. The primers used in the present study are listed in Table 1.

**TABLE 1:** Primers used for in the present study.

Primer name	Sequence (5′ to 3′)	Application
ApPTEN	F: ATG​GCT​TTG​ACT​TGG​ATT​TGA	ORF amplification
	R: ACT​CGT​CGT​CTG​TAT​CGG​TGT	
AiPTEN	F: ATG​GCT​TTG​ACT​TGG​ATT​TGA	ORF amplification
	R: ACT​CGT​CGT​CTG​TAT​CGG​TGT	
RT-ApPTEN	F: TAT​ACA​CTG​TAA​AGC​TGG​AAA​GG	qRT-PCR
	R: TGT​AGT​AAG​GCT​TGG​TTG​TAG​TC	
RT-AiPTEN	F: ACA​CTA​CGG​CCA​GAC​GAG​AAC	qRT-PCR
	R: CAC​ATC​AAA​CAT​CGG​GCT​ACA	
RT-FoxO	F: GGA​AGT​GTT​GCT​CGT​CAG​TCC​TC	qRT-PCR
	R: GCA​CTT​GTT​CCA​TGT​CAC​ATC​CC	
RT-MnSOD	F: AGC​TGA​AGC​AAC​AGA​GAC​AAA​A	qRT-PCR
	R: GGG​CTA​AGA​ACC​TCC​CAG​AAA​A	
RT-CAT	F: CAC​CAA​AAC​AGC​CAC​ACT​AAC​CGC	qRT-PCR
	R: GAC​CTC​AAG​ATA​TCC​AAA​CGC​ACC	
RT-EF1α	F: GAA​AGG​GGC​CTA​TGG​AAT​CGT​AT	qRT-PCR
	R: ATC​TGT​CTG​GTT​TCT​GAA​GGC​AT	
ApPTENi	F: GCU​CAC​CUG​CUG​GUA​GUA​ATT	RNA interference
	R: UUA​CUA​CCA​GCA​GGU​GAG​CTT	

### Sequence Analyses

Identity analysis of the cDNA sequences of *ApPTEN* and *AiPTEN* with known sequences published in NCBI was conducted with BLASTX software online (https://blast.ncbi.nlm.nih.gov/). The open reading frame (ORF) was identified using the ORF finder in NCBI (http://www.ncbi.nlm.nih.gov), and the ORFs of *ApPTEN* and *AiPTEN* were translated into the amino acid sequence using DNAMAN 8.0. The values of Ka, Ks, and Ka/Ks were calculated by the Tbtools software (Chen et al., 2020). The prediction of phosphorylation and glycosylation sites was performed using the NetPhos 2.0 Server (https://services.healthtech.dtu.dk/service.php?NetPhos-3.1) and NetNGlyc 1.0 Server (https://services.healthtech.dtu.dk/service.php?NetNGlyc-1.0), respectively. The functional domains of ApPTEN and AiPTEN were identified using SMART online tool (http://smart.embl-heidelberg.de/). The secondary and three-dimensional structures of ApPTEN and AiPTEN were predicted using SOPMA (http://nhjy.hzau.edu.cn/kech/swxxx/jakj/dianzi/Bioinf7/Expasy/Expasy8.htm) and SWISS-MODEL online tool (https://www.swissmodel.expasy.org/interactive), respectively.

### Sequence Alignment and Phylogenetic Study of the PTEN

Amino acid sequences of PTEN from 10 species were obtained from Genbank database, including *Homo sapiens*, *Mus musculus*, *Rattus norvegicus*, *Danio rerio*, *Pan troglodytes*, *Heterocephalus glaber*, *Orcinus orca*, *Pecten maximus*, *Mizuhopecten yessoensis*, and *Mytilus coruscus*. The mature peptides of PTEN in these species were aligned with the deduced amino acid sequence of ApPTEN and AiPTEN using ClustalW2 (http://www.ebi.ac.uk/Tools/msa/clustalw2/). The aligned sequences were used to construct the phylogenetic tree based on the Neighbor-Joining (NJ) Method of Molecular Evolutionary Genetics Analysis (MEGA 8.0). Bootstrap analysis was repeated 1,000 times to compute a confidence interval.

### Expression Profile and Tissue Distribution of PTEN

In order to analyze the spatial distribution of PTEN in different tissues, 10 healthy individuals from *A. purpuratus* and *A. irradians* were collected. Six tissues (adductor muscle, hepatopancreas, gill, mantle, kidney, and gonad) were dissected separately from each individual of *A. purpuratus* and *A. irradians*. All collected samples were immediately frozen in liquid nitrogen and stored at −80°C for RNA extraction. Total RNA of the samples was separately extracted using the Trizol reagent (TaKaRa, Kusatsu, Japan) and phenol chloroform method. The concentration and purity of the RNA was determined by Nanodrop 2000 spectrophotometer (Nano Drop Technologies, United States), and RNA integrity was detected by electrophoresis on 1.2% agarose gel electrophoresis. Reverse transcription kit ReverTra Ace® qPCR RT Master Mix with gDNA Remover (Toyobo, Japan) was used for synthesis of the cDNA first strand. Taq Pro Universal SYBR qPCR Master Mix (Vazyme Biotech, China) and QuantStudio™ 5 Real-Time PCR Instrument (Thermo Scientific, United States) were used for real-time fluorescence quantification. The reaction system was: 5 μl SYBR® Green Realtime PCR Master Mix, 3.6 μl double-distilled H_2_O, 1 μg cDNA template, 0.2 μl each primer, with a total of 10 μl. *EF1α* was used as an internal control gene and gene specific primers were synthesized according to the previous study (Ning et al., 2021). The primer information of all the genes was shown in Table 1. The reaction was performed under the following conditions: 10 min at 95°C for initial denaturation, and 40 cycles of 10 s at 95°C for denaturation, 15 s at 60°C for annealing, and 15 s at 72°C for extension. Melt curve analysis was carried out over a range from 60 to 94°C at the end of each PCR run. The comparative 2^−ΔΔCT^ method was used to analyze the expression levels of target genes. For each sample, the RT-qPCR was performed with three replications.

### Effects of Nutrition Restriction on Expression of PTEN

To examine the function of PTEN in longevity in response to nutrient restriction, 300 healthy individuals were randomly selected from the *A. purpuratus* and *A. irradians* population and evenly divided into the nutrient restriction group and the control group. During the experiment period, each scallop in the control group were fed with about 3 × 10^4^ cells of *Chaetoceros* sp. per day, while in the nutrition restriction group only a quarter of the diet was fed to the animals. Half of the water in the tank was changed each day. The hepatopancreas of ten healthy individuals from the experimental and the control group were collected on Day 0, Day 30, and Day 56. All collected samples were immediately frozen in liquid nitrogen and stored at −80°C for subsequent RNA extraction and quantification of PTEN expression using the protocol described above.

### Effects of Ionizing Radiation on Expression of PTEN

To examine the function of PTEN in maintenance of genome integrity, 360 healthy individuals were randomly selected and evenly divided into two groups from both the *A. purpuratus* and *A. irradians* populations. For each species, one group of 180 scallops were exposed to ionizing radiation at a dose of 200 Gy for 30 s at the radiation facility of Shandong Lanfu High Energy Physics Technology Co., Ltd., and another group of 180 animals were not treated and used as control. The scallops were then reared in the laboratory at Yantai Institute of Coastal Zone Research, Chinese Academy of Sciences for 7 days to compare the mortality rate between the treatment group and the control group. During the experiment period, all the scallops were fed with *Chaetoceros* sp. three times per day, and half of the water in the tank was changed daily. The adductor muscle, hepatopancreas, gills, and mantles of six individuals from the treatment and the control groups were collected separately at the 48 h in both species. All collected samples were immediately frozen in liquid nitrogen and stored at −80°C for subsequent RNA extraction and quantification of PTEN expression using the protocol described above.

### RNAi of PTEN

To explore the potential pathways for the function of PTEN, RNAi of PTEN was carried out to examine the expressions of the genes downstream to PTEN, including *FoxO*, Mn-Superoxide dismutase (*Mn-SOD*) and catalase (*CAT*). Small interfering RNA (siRNA) was designed basing on the ORF sequence of ApPTEN and synthesized using the T7 promoter *in vitro* transcription kit (Takara, Japan). The synthesized siRNAs were purified and dissolved in DEPC water, and the fragment integrity and concentration of siRNAs were checked by 1.2% agarose gel electrophoresis and Nanodrop 2000 (Thermo Scientific, United States). One hundred healthy Peruvian scallops were randomly selected and evenly divided into RNAi group and control group. In the RNAi group, each animal was injected 10 μL dsRNA with a concentration of 1 μg/g weight into pericardial cavity; while in the control group, same volume of DEPC solution was injected. Gills from six individuals of the RNAi and control group was collected separately at 12, 24, 48, and 72 h. All collected samples were immediately frozen in liquid nitrogen and stored at −80°C for subsequent RNA extraction and quantification of PTEN by RT-qPCR using the protocol described above. After the verification for the effectiveness of the injection and dsRNA, the expressions of *FoxO*, *Mn*-*SOD*, and *CAT* were determined with the primers designed based on the sequences from genomic data after validation.

### Statistical Analyses

As events and RT-qPCR data were expressed as mean ± S.E.M., statistical differences were evaluated by *t*-test and the level of statistically significant difference was set at *p* ≤0.05. All statistics were done with SPSS 19.0 (SPSS, Chicago, United States). The survival analysis of the ionizing radiation group and control group was performed by GraphPad Prism 8.0.2.

## Results

### Molecular Characterization and Bioinformatics Analysis of *ApPTEN* and *AiPTEN*

**FIGURE 1:**
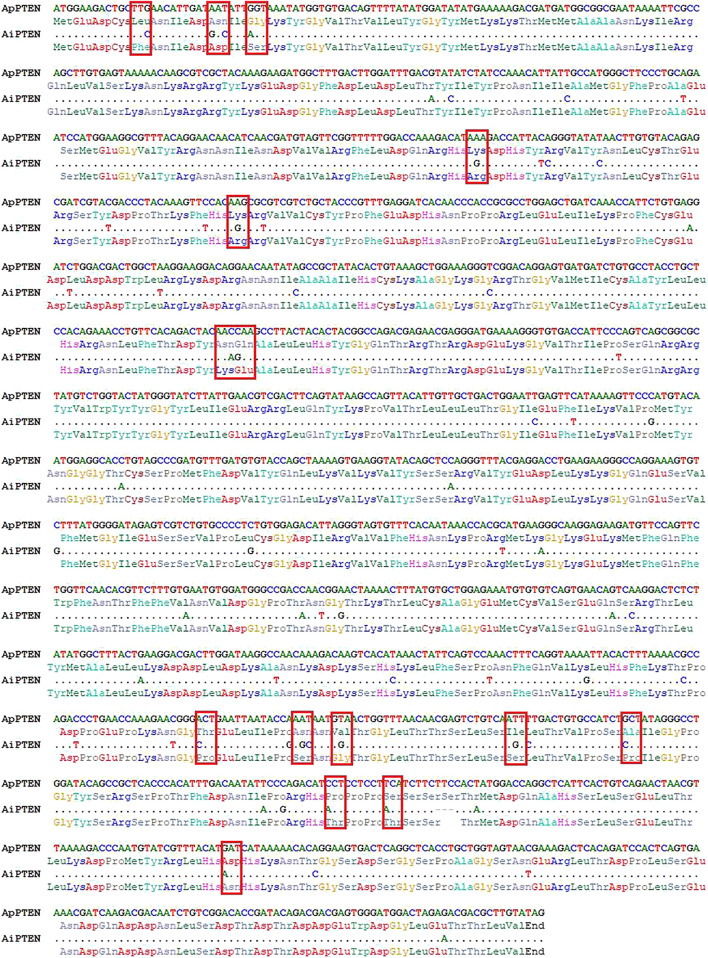
The nucleotide and amino acid variation between ApPTEN and AiPTEN.

Different cDNA sequences were obtained for *ApPTEN* and *AiPTEN* and submitted to the NCBI GenBank under accession nos. OM304359 and OM304360, respectively. The ORFs of the *ApPTEN* and *AiPTEN* were 1,476 and 1,473 bp, encoding 491 and 490 amino acids, respectively. Sixty-four variations were found between the nucleotide sequences of *ApPTEN* and *AiPTEN*, with 48 synonymous and 16 non-synonymous SNPs that resulted in 15 amino acid variation between them (Figure 1). There was one indel with three nucleotides lost in *AiPTEN*, leading to S453 lacked in AiPTEN (Figure 1). The values of Ka, Ks, and Ka/Ks between *ApPTEN* and *AiPTEN* were 0.0133, 0.1683, and 0.0800, respectively.

**FIGURE 2:**
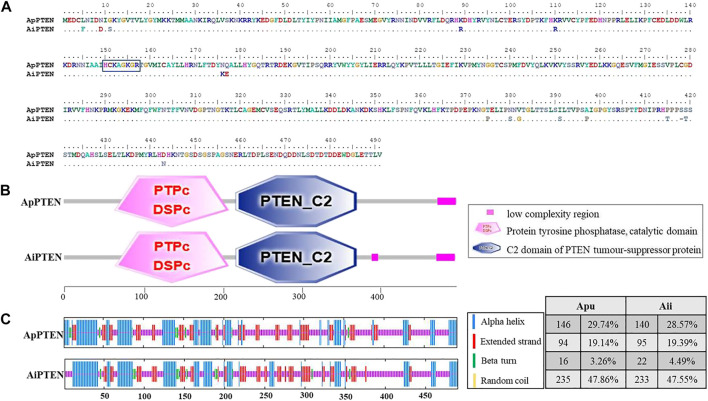
The alignment **(A)**, functional domains **(B)**, and secondary structure **(C)** of the ApPTEN and AiPTEN.

Specific protein phosphorylation sites were predicted from the amino acid sequences of ApPTEN and AiPTEN. In ApPTEN, the potential specific phosphorylation sites included Y92, T393, T407, S418, S420, S453, and T447, while T173, S380, T386, S391, S452, and T415 were the specific phosphorylation sites in AiPTEN (Table 2). The amino acids at the 89, 176, 177, 375, 380, 382, 391, 397, 415, 418, 419, and 443 of ApPTEN were different from AiPETN, among which S380, S391, T415, and S418 were the specific phosphorylation sites and others were near the specific phosphorylation sites (Figure 2A).

**TABLE 2:** The specific phosphorylation sites in ApPTEN and AiPTEN.

	Sites in ApPTEN	Sites in AiPTEN
Serine (S)	418, 420, 453	380, 391, 452
Threonine (T)	393, 407, 447	173, 386, 415
Tyrosine (Y)	92	

In addition, studies have reported that the thermal stability, enzymatic stability, conformational stability, and solubility of proteins are significantly increased after protein glycosylation modifications (Broersen et al., 2004; Jafari-Aghdam et al., 2005). The prediction showed that ApPTEN had four N-glycosylation sites (N314, N373, N381, and N473), but AiPTEN had three N-glycosylation sites (N314, N381, and N472). The amino acid variation at 375aa might affect the glycosylation of N373 in AiPTEN.

### The Structure Analysis of PTEN

SMART analysis showed that there were two functional domains (PTPc_DSPc and PTEN_C2) in ApPTEN and AiPTEN, which located from 64-206aa and 215-366aa, respectively (Figure 2B). The PTPc_DSPc is the catalytic domain of protein tyrosine phosphatase. PTEN_C2 is the C-terminus of the phosphatidyl-inositol triphosphate phosphatase, which associates across an extensive interface with the N-terminal phosphatase domain DSPc. Both the ApPTEN and AiPTEN had a low complexity region at 466-489aa, but the AiPTEN had an extra low complexity region at 385-393aa.

The variations of nucleotides and amino acids for the two domains were analyzed between ApPTEN and AiPTEN. There were 15 SNPs located in the PTPc_DSPc domain, containing 4 non-synonymous SNPs that generated 4 amino acids variation, but there was no variation in the PTEN_C2 domain. A signature motif of HCKAGKGR was found in the PTPc_DSPc domain of both ApPTEN and AiPTEN, although there was a variation of 2 amino acids near the PTPc_DSPc domain between them (Figure 2A). In the PTPc_DSPc domain, the Y92 in ApPTEN and the T173 in AiPTEN were specific phosphorylation sites. In the PTEN_C2 domain, there were an extra N-glycosylation site at N373 in ApPTEN, but not in AiPETN.

Prediction of the secondary structure of PTENs showed that alpha helix, random coil and extended strand were the main form of secondary structure in both species. The ApPTEN was characterized by 30.55% alpha helix, 46.64% random coil, 19.14% extended strand, and 3.67% beta bridge, but AiPTEN was characterized by 28.57% alpha helix, 47.55% random coil, 19.39% extended strand, and 4.49% beta bridge (Figure 2C).

**FIGURE 3:**
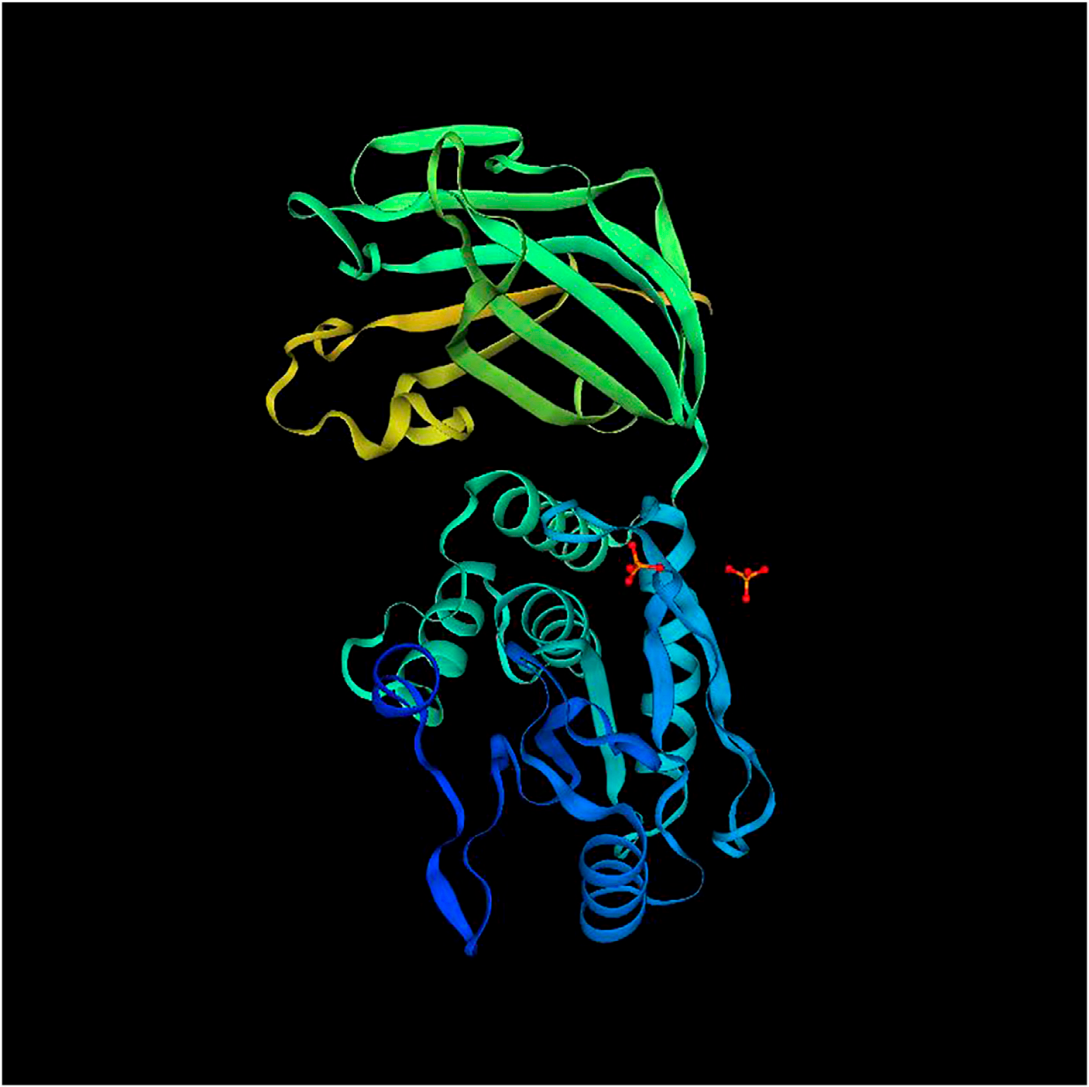
The 3D structure of PTEN (7jvx.1.A).

To elucidate the influence of the difference in primary and secondary structures between ApPTEN and AiPTEN on the conformation, Swiss-Model online tool pair was further used to predict for the 3D structure of ApPTEN and AiPTEN. The results showed that the ApPTEN and AiPTEN were all mapped to crystal structure of 7jvx.1.A (Phosphatidylinositol 3,4,5-trisphosphate 3-phosphatase and dual-specificity protein phosphatase PTEN) with identity of 61.38% and 59.59%, respectively (Figure 3).

### Analysis for the ApPTEN and AiPTEN in the Evolutionary History

**FIGURE 4:**
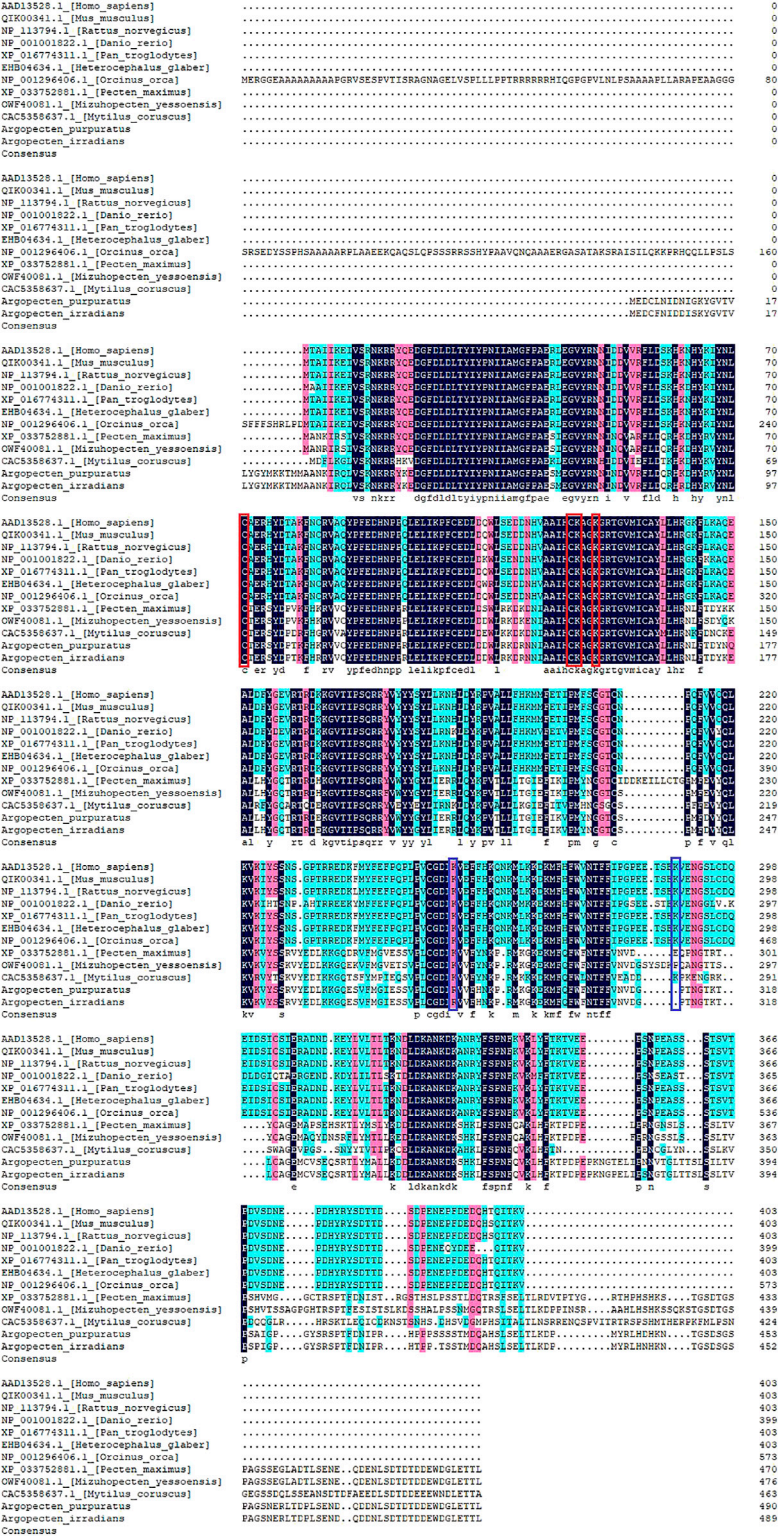
Multiple alignment of PTEN in the 12 species. The conserved and specific modification sites are represented by red and blue frame, respectively.

Multiple alignment of PTEN sequences showed that the domains of PTEN were conserved in the analyzed species. However, the domains of PTEN in molluscs has some variation compared with vertebrates (Figure 4). Meanwhile, the post-translational modification sites of C98, C151, K152, and K155 were conserved across all species, except the sites of K255 and K289 in ApPTEN and ApPTEN, suggesting that they may be central sites for the protein activity (Chen et al., 2018) (Figure 4).

**FIGURE 5:**
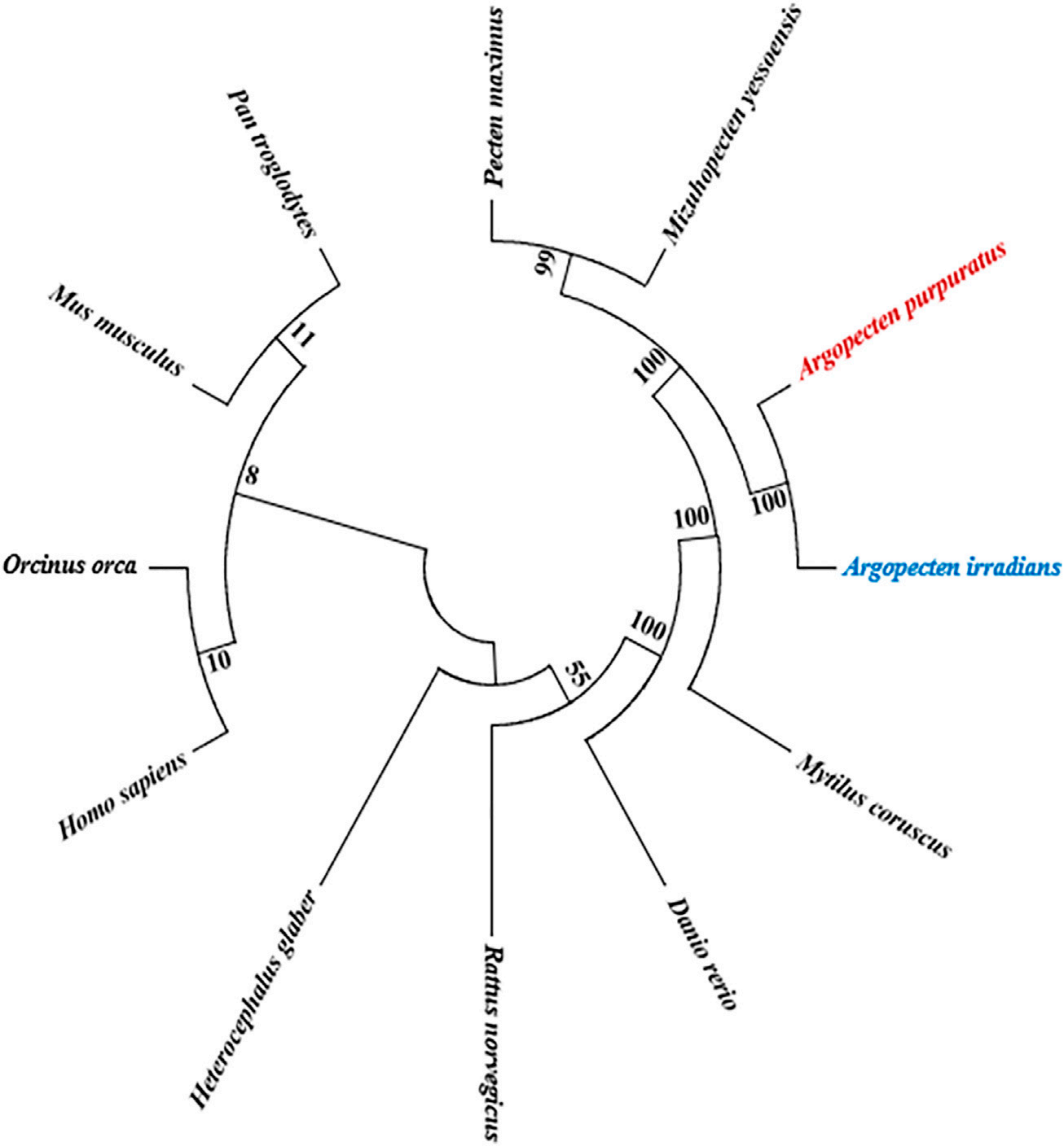
NJ phylogenetic analysis of ApPTEN and *AiPTEN* with other species.

Phylogenetic tree showed that AiPTEN and ApPTEN were closely related to *M. yessoensis*, *P. maximus* and clustered into one branch, followed by *M. coruscus*, but far related to *H. glaber*, *H. Sapiens*, *O. orca*, and *P. troglodytes*, which have longer life span than bivalves (Figure 5).

### Expression Pattern of *ApPTEN* and *AiPTEN* in Different Tissues

**FIGURE 6:**
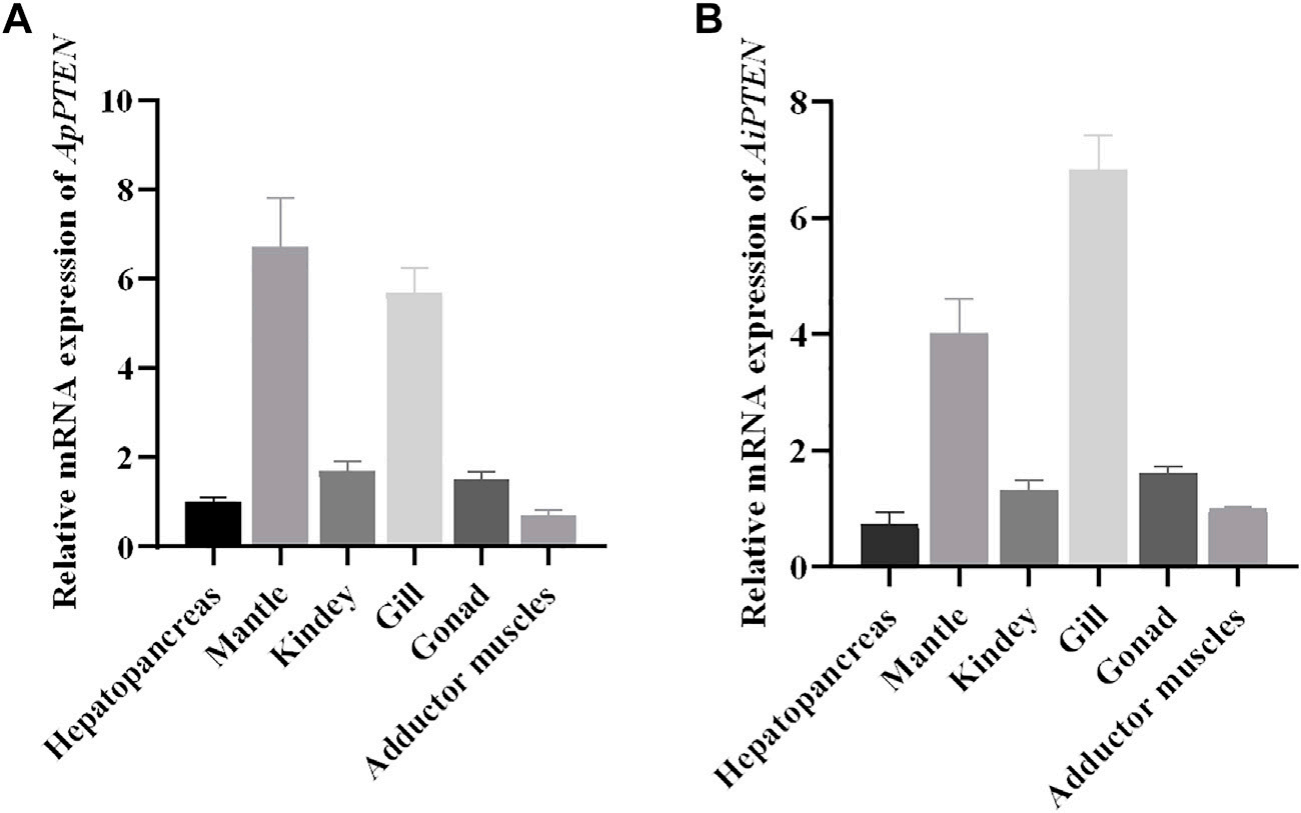
Relative mRNA expressions of *ApPTEN* **(A)** and *AiPTEN* **(B)** in different tissues.

The mRNA expression profile of *PTEN* in the hepatopancreas, mantle, kidney, gill, gonad, and adductor muscle were similar in *A. purpuratus* and *A. irradians* (Figure 6). *PTEN* was expressed in all examined tissues, with higher expression in mantle and gill and lower expression in the adductor muscle, kidney, hepatopancreas, and gonad.

### Expression of *ApPTEN* and *AiPTEN* Under Nutrient Restriction

**FIGURE 7:**
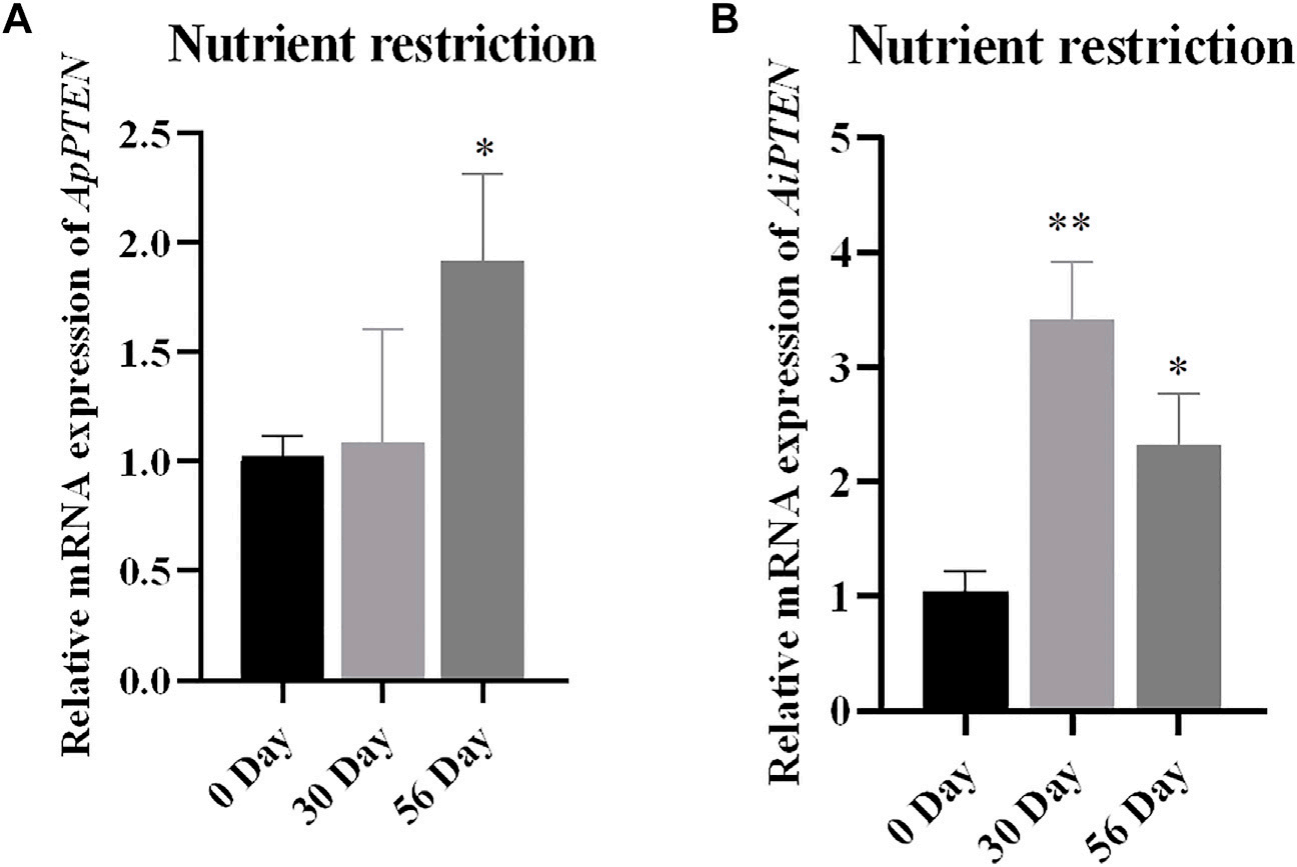
Relative mRNA expressions of *ApPTEN* **(A)** and *AiPTEN* **(B)** in hepatopancreas under.

Under nutrient restriction, the expression of *PTEN* was up-regulated in *A. purpuratus* and *A. irradians*, but the response in *A. irradians* was faster and more robust than in *A. purpuratus* (Figure 7)*.* As can be seen in Figure 7, expression of *PTEN* was significantly increased at Day 30 (*p* < 0.01) and remained higher at Day 56 than that of Day 0 (*p* < 0.05) in *A. irradians*, but only significantly elevated at Day 56 under nutrient restriction in *A. purpuratus*.

### Expression of *ApPTEN* and *AiPTEN* After Ionizing Radiation

**FIGURE 8:**
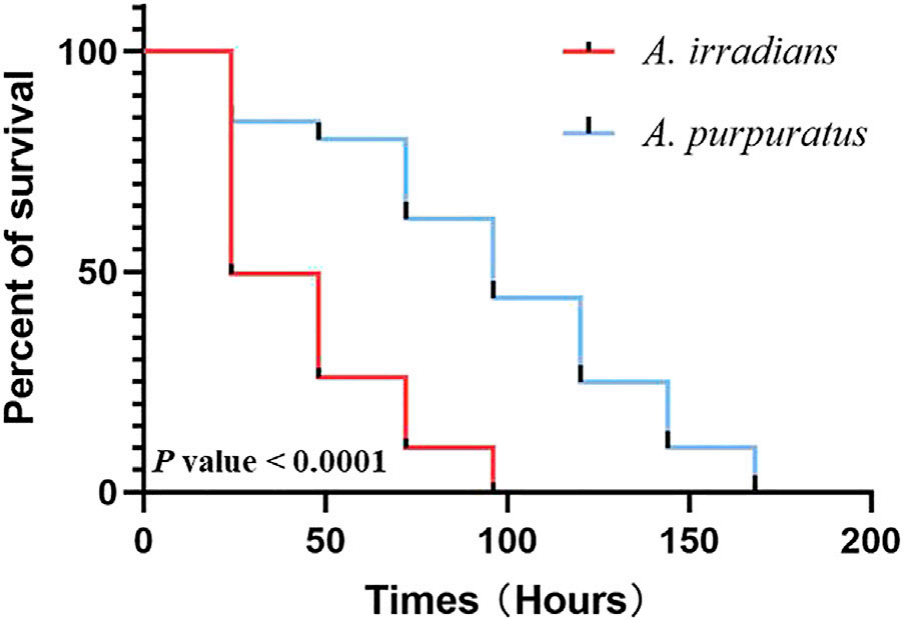
The survival curve of *A. purpuratus* and *A. irradians* after ionizing radiation.

**FIGURE 9:**
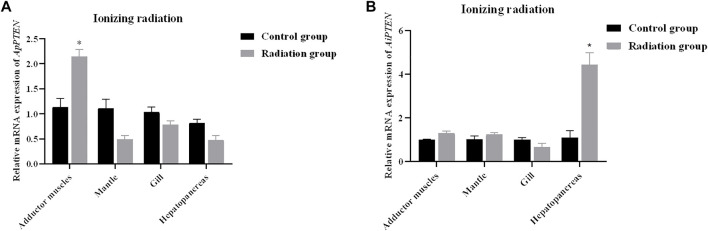
Relative mRNA expressions of *ApPTEN* **(A)** and *AiPTEN* **(B)** after ionizing radiation (*p < 0.05).

After ionizing radiation, the survival rate of *A. purpuratus* was significantly higher than that of *A. irradians* (Figure 8). Forty-eight hours after ionizing radiation, the expression of *PTNE* gene was significantly increased in adductor muscles of *A. purpuratus* and in hepatopancreas of *A. irradians* in the ionizing radiation group (*p* < 0.05; Figure 9).

### Expression of the Downstream Genes Under Inhibiting PTEN

**FIGURE 10:**
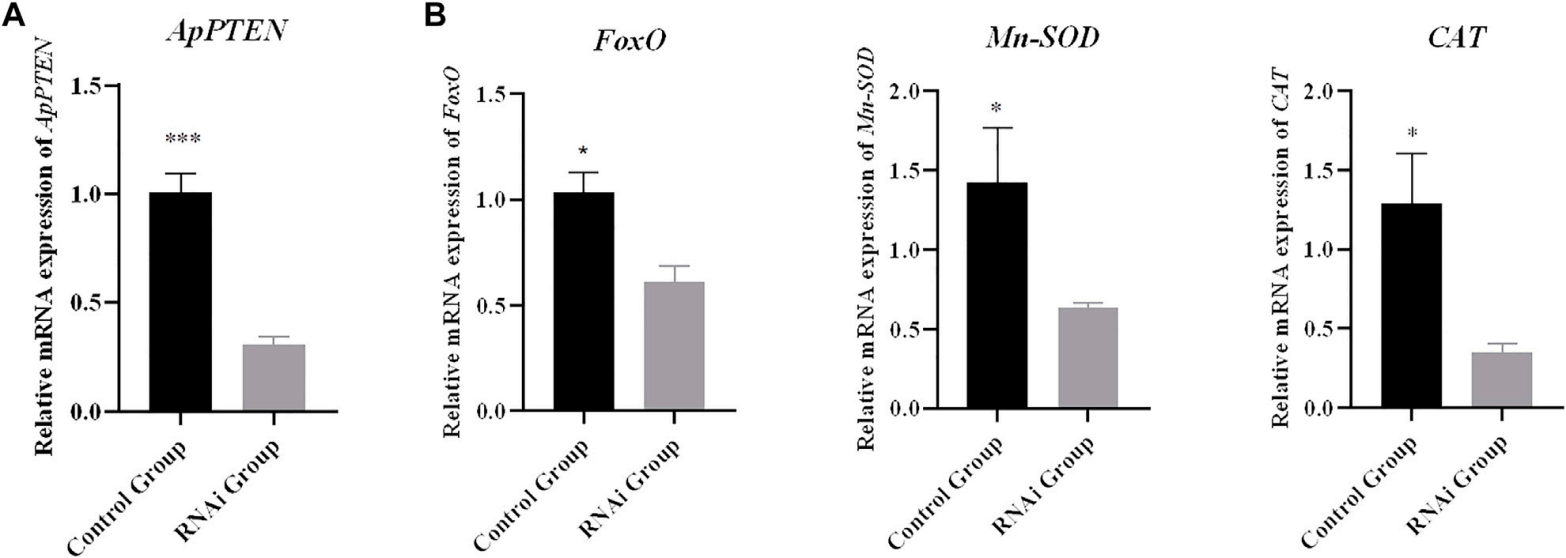
Relative mRNA expressions of target genes **(B)** after inhibition of *ApPTEN* **(A)** expression (*p < 0.05 and ***p < 0.001).

After 12 h of RNA interference, the mRNA expression of *PTEN* was significantly decreased in the RNAi group compared with the control group (*p* < 0.05), with an interference efficiency of 69.7% (Figure 10A). The expression levels of *FoxO*, *Mn-SOD,* and *CAT* were significantly decreased in RNAi group compared with the control group (*p* < 0.05) (Figure 10B).

## Discussion

Studies in terrestrial model organisms such as nematode worms, flies, and mice have shown that PTEN is a unique negative regulator of the PI3K/Akt/FoxO signaling pathway, which plays a central role in a series of biological processes such as cancer protection, metabolism, apoptosis, and longevity. However, until now there was no report on PTEN in marine organisms. In the present study, we cloned and characterized the *PTEN* in two closely related scallops, *A. purpuratus* (*ApPTEN*) and *A. irradians* (*AiPTEN*), which have significantly different life spans. We then investigated the roles of PTEN in the modification of longevity in these animals.

Similar sequences of PTEN were found in two scallops, except 48 synonymous and 16 non-synonymous SNPs and one InDel that resulted in a loss of S453 in *AiPTEN*. Previous studies have uncovered that synonymous and non-synonymous substitutions can significantly alter protein function through a wide variety of mechanisms, such as protein level, translational accuracy, secretion efficiency, the final folded structure, and post-translational modifications (Tuller et al., 2010; Walsh et al., 2020). The mutations in sequence may lead to changes in the secondary structure and tertiary conformation of PTENs, suggesting that PTEN in the two scallops may have evolved into different conformations in adaptation to different environments (Drummond and Wilke, 2008). The value of Ka/Ks between ApPTEN and AiPTEN (0.0800) indicated this gene may have been subjected to purifying selection in these two scallops. The signature motif HCxxGxxR of PTEN was essential for its catalysis and phosphorylation activity (Chen and Guo, 2017), and the variation of two amino acids between ApPTEN and AiPTEN might lead to different catalytic activity of PTEN in the two species. There were 7 and 6 specific phosphorylation sites in ApPTEN and AiPTEN, respectively, with the S453 lost in AiPTEN. Phosphorylation is a widespread post-translational modification of proteins and plays an important role in the regulation of protein activity through conformation changes (Cohen, 2002; Kim et al., 2004). Several enzymes are responsible for the phosphorylation of PTEN, including casein kinase 2 (CK2), glycogen synthase kinase-3 (GSK3β), RhoA kinase, and P110δ subunits of PI3K (Chen et al., 2018). The variations in phosphorylation sites of PTEN may affect the activity of PTEN (Torres and Pulido, 2001; Torres et al., 2003; Al-Khouri et al., 2005). In addition, we found that glycosylation occurred more frequently in ApPTEN than AiPTEN. Glycosylation modification can not only alter the stability of protein, but also the biological activity of protein molecules (Zachara and Hart, 2002; Oka et al., 2004; Blawid et al., 2007). Therefore, we deduced that the different phosphorylation and glycosylation sites might contribute to the different activities in ApPTEN and AiPTEN. Therefore, the key phosphorylation and glycosylation sites should be further studied in future research.

Although the sequences of ApPTEN and AiPTEN are slightly different from those of the model organisms, they contained the primary functional domains that were conserved throughout the animal kingdom, including PTPc_DSPc and PTEN_C2 (Chen et al., 2018). These results suggest that PTEN in the scallops might have similar physiological functions as in other animals. However, the mutation at some key posttranslational modification sites in the scallops suggested PTEN might have different activity from other species with longer life span (Figure 4). In the present study, PTEN expressed in all examined tissues, indicating it was widely expressed and not a tissue specific gene. However, higher expression was detected in the mantles and gills. The mantle is the main tissue for fabrication of molluscan shell by its specialized epithelial cells (McDougall and Degnan, 2018), indicating PTEN played an important role in metabolism. In bivalves, the gills are the dominant site of interaction with the environment, mediated by the creation of water currents in the mantle cavity (Cannuel et al., 2009). Thus, PTEN may have a positive role in protecting scallops against adverse effects of environmental stress.

It has been found in the model organisms that PTEN may be involved in the regulation of longevity through several ways, such as regulation of energy availability, genomic integrity, and oxidative damage caused by ROS (Barja, 2013; Carmona and Michan, 2016; Lian et al., 2019). In the present study, we found the mRNA expression of *ApPTEN* and *AiPTEN* under starvation was significantly increased, and the response in *A. irradians* was faster and more robust than in *A. purpuratus.* It seems that PTEN might play a crucial role in the physiological response of the scallops to nutrient availability, as starvation stress leads to an overall decrease in metabolic activity, with reduced lipid metabolism, protein biosynthesis, protein hydrolysis, cellular respiration, and increased glucose xenobiotic activity (Drew et al., 2008; Grone et al., 2012). The faster and more robust response in *A. irradians* than in *A. purpuratus* might be resulted from their long-term adaptation to different environments. The Peruvian scallop *A. purpuratus* is cold-water species, which might experience food shortage and become less sensitive to nutritional restriction. The bay scallop *A. irradians*, on the contrary, is a warm water species (DiSalvo et al., 1984) and may have a higher demand for food and becomes more sensitive to starvation. In previous studies, it was reported that cold water temperatures and presumably food limitations might be favorable for longevity of bivalves (Buick and Ivany, 2004; Abele et al., 2009). Consequently, we speculate that PTEN might also have the conserved function on longevity by responding to nutrient availability in *A. purpuratus*.

Genome instability has long been implicated as the main causal factor in aging (Carmona and Michan, 2016), and low doses of radiation shorten life span and accelerate the accumulation of pathological lesions in late age (Vijg and Suh, 2013). In the present study, the survival of *A. purpuratus* was significantly higher than *A. irradians* after ionizing radiation, indicating *A. purpuratus* has higher ability of DNA damage repair to maintain the genome integrity. Ionizing radiation significantly elevated the expression of *PTEN* in the adductor muscles of *A. purpuratus* but not *A. irradians*, indicating PTEN might be involved in DNA damage repair in *A. purpuratus*. These results may lend support for the positive actions of PTEN in the longevity in the Peruvian scallops.

ROS released by electron transport systems of the mitochondria and the endoplasmic reticulum is found to contribute to aging (Bratic and Larsson, 2013). The antioxidant systems, especially SOD and CAT, play an important role in controlling ROS at a low level (Pomatto and Davies, 2018). According to the reports in model organisms, the transcription factor FoxO is an important downstream gene regulated by PTEN through PI3K/Akt pathway, and it was proved to positively regulate longevity by increasing the antioxidant activities of its target genes *Mn-SOD* and *CAT* (Martins et al., 2016). In the PTEN/PI3K/Akt/FoxO pathway, PI3K/AKT phosphorylates FoxO to inhibit its activity (Gross et al., 2009), and PTEN as the irreplaceable factor inhibits the PI3K/AKT signaling pathway by converting PIP3 to PIP2 (Wishart and Dixon, 2002). In the present study, the results showed that the expression of FoxO, Mn-SOD, and CAT were all significantly downregulated by PTEN-RNAi. The ocean quahog *Arctica islandica*, a bivalve regarded as a prototype example for negligible senescence (lifespan approaching 400 years), is known to maintain high antioxidant activities in gills and mantles by SOD and CAT (Abele et al., 2009), and its post-maturation antioxidant activity level was found to be 10-fold over other bivalve species with shorter life span (Abele et al., 2009). Our results suggested that the PTEN/PI3K/Akt/FoxO pathway might also be conserved in aquatic animals and related to the longer life span of *A. purpuratus*. Further exploration on how this pathway modify the longevity in the scallops are certainly warranted in future studies.

## Conclusion

The fundamental mechanism of aging and longevity has been a worldwide hot issue, and existing studies mainly focused in several terrestrial model organisms, but with only a few reports in aquatic animals. Some scholars had suggested that bivalves may be more appropriate for aging and longevity studies which may help explore the genetic basis of the lifespan in higher animals. We cloned and characterized the *PTEN* in two closely related scallops that have significantly different life spans, and investigated its function on longevity in these scallops. The results indicated that PTEN mutated at some sites and resulted in conformation change, which may be a result of long-term adaptations to different environments in these two species. In addition, the results suggested, as in the model organisms, PTEN also play important role in modification of longevity through its response to nutrient availability, genomic stability, and controlling of ROS *via* PTEN/PI3K/Akt/FoxO pathway. The results of the present study may provide important information for understanding of the conserved function of PTEN in longevity of animals and human. It may also benefit the selection of scallops strains with long lifespan and thus larger size.

## Data Availability

The datasets presented in this study can be found in online repositories. The names of the repository/repositories and accession number(s) can be found in the article/Supplementary Material.
